# Laparoscopic liver resection for non-colorectal non-neuroendocrine metastases: perioperative and oncologic outcomes

**DOI:** 10.1186/s12957-019-1700-y

**Published:** 2019-09-04

**Authors:** Davit L. Aghayan, Piotr Kalinowski, Airazat M. Kazaryan, Åsmund Avdem Fretland, Mushegh A. Sahakyan, Bård I. Røsok, Egidijus Pelanis, Bjørn Atle Bjørnbeth, Bjørn Edwin

**Affiliations:** 1 The Intervention Centre, Oslo University Hospital—Rikshospitalet, Pb. 4950 Nydalen, 0424 Oslo, Norway; 2 Institute of Clinical Medicine, Faculty of Medicine, University of Oslo, Oslo, Norway; 3 Department of Surgery N1, Yerevan State Medical University after M. Heratsi, Yerevan, Armenia; 4 Department of General, Transplant and Liver Surgery, Medical University of Warsaw, Warsaw, Poland; 5 Department of Surgery, Fonna Hospital Trust, Stord, Norway; 6 Department of Faculty Surgery N2, I.M.Sechenov First Moscow State Medical University, Moscow, Russia; 7 Department of Hepato-Pancreato-Biliary Surgery, Oslo University Hospital, Oslo, Norway; 8 Department of General and Laparoscopic Surgery, Central Clinical Military Hospital, Yerevan, Armenia

**Keywords:** Laparoscopic liver surgery, Non-colorectal, Non-neuroendocrine liver metastases, Survival

## Abstract

**Background:**

Liver resection is a treatment of choice for colorectal and neuroendocrine liver metastases, and laparoscopy is an accepted approach for surgical treatment of these patients. The role of liver resection for patients with non-colorectal non-neuroendocrine liver metastases (NCNNLM), however, is still disputable. Outcomes of laparoscopic liver resection for this group of patients have not been analyzed.

**Material and methods:**

In this retrospective study, patients who underwent laparoscopic liver resection for NCNNLM at Oslo University Hospital between April 2000 and January 2018 were analyzed. Perioperative and oncologic data of these patients were examined. Postoperative morbidity was classified using the Accordion classification. Kaplan–Meier method was used for survival analysis. Median follow-up was 26 (IQR, 12–41) months.

**Results:**

Fifty-one patients were identified from a prospectively collected database. The histology of primary tumors was classified as adenocarcinoma (*n* = 16), sarcoma (*n* = 4), squamous cell carcinoma (*n* = 4), melanoma (*n* = 16), gastrointestinal stromal tumor (*n* = 9), and adrenocortical carcinoma (*n* = 2). The median operative time was 147 (IQR, 95–225) min, while the median blood loss was 200 (IQR, 50–500) ml. Nine (18%) patients experienced postoperative complications. There was no 90-day mortality in this study. Thirty-five (68%) patients developed disease recurrence or progression. Seven (14%) patients underwent repeat surgical procedure for recurrent liver metastases. One-, three-, and five-year overall survival rates were 85%, 52%, and 38%, respectively. The median overall survival was 37 (95%CI, 25 to 49) months.

**Conclusion:**

Laparoscopic liver resection for NCNNLM results in good outcomes and should be considered in patients selected for surgical treatment.

## Introduction

Patients with NCNNLM constitute a highly heterogeneous group in terms of primary tumor location, biology, mechanisms of spread, and treatment outcomes [1].

Surgical resection is considered the only potentially curative treatment for resectable colorectal liver metastases (CRLM), with 5-year survival rates following resection between 30 and 58%, which is superior to medical therapy only [2–4]. Hepatic resection provides a supreme opportunity for long-term survival in patients with metastatic neuroendocrine tumors and is considered the only curative option for this group of patients [5].

The encouraging results for liver resection for CRLM are not easily transferable to NCNNLM due to scarcity and low quality of publications. However, early reports on liver resection for non-colorectal liver metastases suggested a favorable outcome in this group of patients [6]. Subsequent studies reporting outcomes of liver resection in patients with NCNNLM identified predictive factors, and a risk model for prognosis was created to help identify patients who may benefit the most from the surgical treatment. Selected groups of NCNNLM patients have outcomes comparable to patients with metastases of colorectal and neuroendocrine origin [1, 7, 8].

Adam et al. [7] designed a prognostic scoring model including factors associated with poor prognosis such as extrahepatic metastases present prior to or at the time of hepatectomy, primary tumor site (non-breast origin), patient age (> 60), short (< 12 months) disease-free interval, major hepatectomy, R2 resection, and histology (melanoma or squamous).

Some of these prognostic factors are related to the technique of liver resection, and a laparoscopic approach may influence their validity in prediction of outcomes. We therefore aimed to study the short- and long-term outcomes after laparoscopic liver resection (LLR) for NCNNLM.

## Methods

### Patients and perioperative management

Oslo University Hospital is a referral center for hepato-pancreato-biliary surgery for South-Eastern Norway Regional Health Authority. Patients, who underwent LLR for NCNNLM between April 2000 and January 2018, were identified from the continuously collected database of laparoscopic liver surgery and included in this study, following the institutional review board approval. All patients were operated on with a radical intent, i.e., one aimed to resect the entire tumor burden in the liver. Four patients with metastases from gastrointestinal stromal tumor were operated on with the intent of removing all active tumor sites, i.e., removal of tumors showing signs of sunitinib resistance during ongoing treatment.

Standard preoperative investigations included clinical biochemistry, liver ultrasound, contrast-enhanced X-ray computed tomography (CT) scans and/or magnetic resonance imaging (MRI) of the thorax and abdomen, and positron emission tomography (PET) scan (if required).

Patients with resectable or stable extrahepatic disease were considered for liver surgery. Surgical technique for LLR at our center has been described previously [9–11]. Laparoscopic ultrasonography and presence of a range of advanced laparoscopic equipment were the prerequisites for LLR and have been described in our previous studies [12, 13].

Non-steroidal anti-inflammatory drugs and intravenous paracetamol were used for postoperative analgesia. Opioids were given if additional analgesia was required. Patients were encouraged to mobilize early and resume oral intake as soon as tolerated.

Patients were treated with neoadjuvant and adjuvant chemotherapy following national guidelines for each specific tumor entity.

### Definitions

The Accordion classification was used to grade postoperative complications [14, 15].

Tumor size was measured following specimen fixation in formaldehyde during the histopathologic analyses of resected specimens. The distance from the tumor to the resection plane was measured macroscopically and microscopically after fixation. All resection margins were assessed microscopically, a resection margin of less than 1 mm was defined as positive (R1). The narrowest resection margin was recorded in cases with multiple concomitant liver resections.

The term “synchronous” was defined as liver metastases detected within 12 months of diagnosis of the primary tumor; otherwise< metastases were defined as “metachronous.” Disease-free interval (DFI) was defined as a time between diagnoses of the primary tumor and liver metastasis. Overall survival (OS) was estimated from the time of liver resection until death, and recurrence-free survival (RFS) was estimated from the time of liver resection until the first registered disease recurrence. Patients with unresectable extrahepatic diseases were excluded from RFS analysis, as well as patients with residual non-active liver metastases.

### Statistics

The data are presented as median (interquartile range) and/or number (percentage). Survival probabilities were calculated using the Kaplan–Meier method, and Adam’s scoring system [7] was used to calculate predicted 5-year survival.

## Results

### Perioperative data

A total of 51 patients underwent LLR for NCNNLM (Table 1). Types of primary tumors are shown in Table 2. Single liver resections were done in 36 patients, two concomitant resections in 10 patients, and three and four concomitant resections in three and two patients, respectively. In total 73 specimens were resected.

**Fig. 1:**
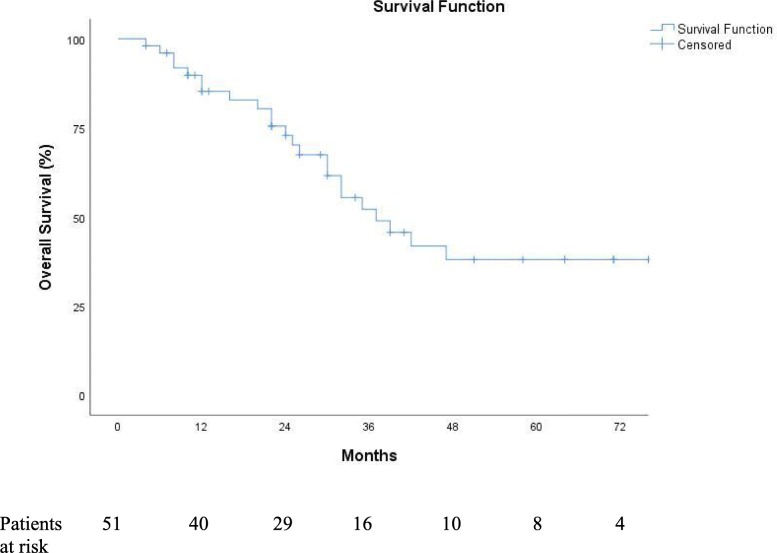
Kaplan–Meier curve for overall survival

**Table 1:** Baseline characteristics

Variable	*N* = 51
Age, years (IQR)	63 (55–70)
Gender (female/male), *n* (%)	29 (57)/22 (43)
BMI	25.6 (16–42.6)
ASA score (%)	
2003ASA I	3 (6)
ASA II	35 (69)
ASA III	13 (25)
Synchronous/metachronous metastases, *n* (%)	21 (41)/30 (59)
Liver involvment, unilobar/bilobar, *n* (%)	38 (74)/13 (26)
Disease free interval, months (IQR)	16 (0–51)
Extrahepatic disease, *n* (%)	16 (31)

*ASA* American Society of Anesthesiology, *BMI* body mass index*, IQR* interquartile range

**Table 2:** Primary tumor diagnosis (*N* = 51)

Groups and diagnosis	No (%)	Neoadjuvant chemo (no, %*)	Adjuvant chemo (no, %*)	5-year OS, %
GI	20 (39)	12 (60)	11 (55)	39
GIST (stomach, intestine)	9	8	8	
Pancreas ca	4	1	2	
Duodenum ca	2	1	–	
Esophagus ca	2	–	–	
Anal ca	3	2	1	
GU	7 (14)	4 (57)	6 (86)	43
Cervix ca	2	2	2	
Endometrial ca	2	–	1	
Uterine sarcoma	2	1	2	
Ovarian ca	1	1	1	
Melanoma	16 (31)	1 (6)	7 (44)	12
Ocular	12	1	5	
Cutaneous	3	–	2	
Unknown melanoma	1	–	–	
Breast c-r	2 (4)	1 (50)	2 (100)	NA
Others	6 (12)	0 (0)	3 (50)	75
Adrenocortical ca	2	–	2	
Retroperitoneal sarcoma	2	–	–	
Lung ca	1	–	1	
Unknown**	1	–	–	
Total	51	18 (35)	29 (57)	38

*NA* not appropriate

*% within the group

**Metastasis from adenocarcinoma, but primary tumor was not found

A total of forty-eight patients underwent parenchyma-sparing liver resection, while three patients had formal liver resection (two left and one right hemihepatectomy).

Three procedures (6%) were converted to open surgery due to hemorrhage (*n* = 1), uncertainty concerning surgical margins (*n* = 1), and duodenal perforation (*n* = 1). In six cases, LLR were combined with liver ablation and in one case with ablation of a concomitant renal metastasis. Seven patients underwent an additional synchronous surgical procedure: two cholecystectomies, one combined cholecystectomy and splenectomy, one repair of a ventral hernia, one resection of the diaphragm, one adrenalectomy, and one removal of a pelvic tumor.

Median operative time was 147 min (95–225), while median blood loss was 200 ml (50–500). Postoperative complications developed in 9 (18%) patients, and there was no 90-day mortality in this study (Table 3). The median hospital stay was 3 days (2–5).

**Table 3:** Perioperative outcomes and histopathological data

Variable	*N* = 51
Operation time, min (IQR)	147 (95–225)
Blood loss, ml (IQR)	200 (50–500)
Number of resections, *n*	73
Combined radiofrequency ablation, *n* (%)	7 (14)
Simultaneous surgical procedures, *n* (%)	7 (14)
Conversion, *n* (%)	3 (6)
Postoperative complications (Grade ≥ 2)	
Total, *n* (%)	9 (18)
Grade 2	4
Small pulmonary embolism	3
Protracted migrane (required opioid)	1
Grade 3	3
Abscess in lower abdomen	1
Infected fluid collection	2
Grade 4	2
Perforated gallbladder, peritonitis	1
Wound dehiscence	1
Length of stay, days, median (range)	3 (2–5)
Readmission, *n* (%)	3 (6)
Resected specimens, *n*	73
Removed lesions, *n*	84
Maximum tumor size, mm, median (IQR)	27 (15–46)
Resection margin, mm, median (IQR)	2 (1–5)
R0/R1/R2* *n*, (%)	33 (65)/14 (27)/4 (8)

*IQR* interquartile range

*Classification as R2 was based on the presence of residual but stable GIST metastases not considered for resection

A total of 84 lesions with a median diameter of 27 mm (15–46) were removed. Median resection margin was 2 mm (1–5) (Table 3).

### Long-term outcomes

Median observation time was 26 months (12–41). Sixteen patients (31%) had extrahepatic disease at the time of liver resection (Table 1). A total of 18 (35%) patients received neoadjuvant chemotherapy, and 29 (57%) patients received adjuvant chemotherapy (Table 2).

Disease recurrence or progression of extrahepatic metastases occurred in 35 (68%) patients. Recurrence in the liver occurred in 28 (55%) patients, including seven patients (14%) who had isolated liver recurrence and one patient (2%) who had recurrence in the resection bed. Seven patients underwent repeat liver surgery including four laparoscopic and two open liver resections and one radiofrequency ablation (Table 4).

**Table 4:** Long-term outcomes

Variable	*N* = 51
Disease recurrence, *n* (%)	35 (68)
Liver recurrence, *n* (%)	28 (55)
Only-liver recurrence, *n* (%)	7 (14)
Recurrence in resection bed, *n* (%)	1 (2)
Repeat surgical procedure for liver recurrence, *n* (%)	7 (14)
Laparoscopic liver resection, *n*	4
Open liver resection, *n*	2
Radiofrequency ablation, *n*	1
Median overall survival, months (95% CI)	37 (25–49)
1-year overall survival rate, %	85 ± 5.2
3-year overall survival rate, %	52 ± 8.2
5-year overall survival rate, %	38 ± 8.6
Recurrence-free survival	*N* = 42
1-year recurrence-free survival rate, %	42 ± 8
3-year recurrence-free survival rate, %	22 ± 7.3
5-year recurrence-free survival rate, %	22 ± 7.3

*CI* confidence interval

Median overall survival was 37 months (95% CI, 25 to 49). One-, three-, and five-year overall survival rates were 85%, 52%, and 38%, respectively (Fig. 1).

One-, three-, and five-year recurrence-free survival was 42%, 22%, and 22%, while the median recurrence-free survival was 9 months (95% CI, 5.6 to 12.4) (Table 4).

## Discussion

In this study, we report short- and long-term outcomes after LLR of NCNNLM. We found perioperative outcomes comparable to previous reports on open liver resection (OLR) as well as outcomes after LLR for CRLM [12, 16, 17] and neuroendocrine tumor (NET) metastases [18, 19]. In our series, 5-year OS in the whole group was 38% which was better than the predicted survival (19%) based on the Adam’s score (Table 5).

**Table 5:** Groups by histological type and Adam score (*N* = 51)

Variable	*N* = 51	5-year OS (%)
Histological type		
Stromal	9	53
Adenocarcinoma	16	49
Sarcoma	4	37
Melanoma	16	12
Squamous cell	4	50
Adrenocortical carcinoma	2	NA
Adam’s Score		
0–3	12 (23)	76/36*
4–6	37 (72)	26/19
7–10	2 (5)	NA

*NA* not appropriate

*5-year overall survival and predicted survival based on Adam’s scoring model

Patients with NCNNLM have traditionally not been referred for evaluation for liver surgery due to a presumed poor prognosis of stage IV disease. Also, a high proportion of these patients had extrahepatic metastases commonly considered a contraindication for liver resection. Recent studies, however, show that more patients with NCNNLM are being considered for liver resection [20, 21]. Despite a trend towards LLR [22], these patients are still mostly considered for OLR [23]. Small series of patients undergoing LLR for NCNNLM have been reported in publications showing similar outcomes of LLR for other metastases [17, 24]. Detailed information on outcomes in this group is rarely presented; therefore, comparisons are limited to published outcomes of OLR for NCNNLM.

To our best knowledge, there is no study reporting short- and long-term outcomes of LLR for this group of patients. The advantages of laparoscopy over its open counterpart are well known [25]. LLR is an established surgical approach for patients with primary liver malignancies [26, 27] and liver metastases of colorectal [13, 25] and neuroendocrine origin [18].

In a recently published randomized controlled trial (OSLO-COMET trial) from our institution comparing OLR and LLR for CRLM [25], we found significantly less postoperative complications following laparoscopy while there were no differences in perioperative outcomes. In the current series, the median operative time was 147 min, while the median blood loss was 200 ml. Postoperative complications developed in 9 patients (17.3%), and median hospital stay was 3 days. Three procedures (6%) were converted to open surgery. These surgical outcomes are comparable to/or better than earlier reported OLR for NCNNLM [23] as well as outcomes of LLR reported for CRLM [17] and NET metastases [18].

Patients with metastases from gastrointestinal (GI) tract represented the largest group (20 patients, 39%) in our cohort followed by melanoma (16 patients, 31%), genitourinary (GU) (7 patients, 14%), and others (6 patients, 12%) but only 2 patients (4%) with metastases from breast cancer (BCLM) (Table 2). In the largest original multicenter report published by Sano et al. presenting results of 1639 liver resections in 1539 patients ,the most common indications for hepatectomy were metastases from gastric cancer (35%), GIST (13%), biliary (10%), ovarian (7%), and pancreatic cancer (5%) [28]. In the second largest study of 1452 patients, BCLM represented 31% of the cohort followed by GU (23%), GI (22%), and melanoma (10%) [7]. In a systematic review of 73 studies involving 3596 patients who underwent liver resection for NCNNLM, the largest group were patients with BCLM (28.2%) followed by GI (19%), GU (15.3%), and broad category of others (30%) including melanoma (18%), sarcoma (5%), and GIST (3%) [20]. There are differences in published studies in proportion of individual diagnoses in the NCNNLM group [28–31].

The heterogeneity of this group and various definitions of factors used in analyses complicate assessment of outcomes. In one of the first reports analyzing factors influencing long-term outcomes of hepatic resection for NCNNLM, Harrison et al. [6] found the primary tumor type of GU origin, more than 36 months of disease-free interval and curative resection predicted longer survival in this group. In a review of open liver resections for NCNNLM, Fitzgerald et al. [20] reported the longest median survival for metastases of GU origin (63.4 months) followed by BCLM (44.4 months).

Based on preoperative factors and outcomes of surgical treatment, Adam et al. [7] designed a scoring system (from 0 to 10) to estimate survival of patients with NCNNLM. In this study, 5-year OS was 38% while the prediction of 5-year OS based on the Adam scoring model was 19% (Table 5). This can be considered a favorable result considering the high proportion of GI and melanoma metastases, which negatively influence prognosis, and the low number of BCLM, which usually have better outcomes (Table 2). Our experience with regard to LLR for melanoma liver metastases has been recently published [32].

The Adam scoring model was created to assess outcomes of patients with NCNNLM grouped together. The outcomes predicted by the model are biased by factors related to various types of tumors. In a recently published large multicenter analysis by Sano et al., Adam’s model was also used and the comparison of outcomes reported. However, Sano et al. showed another source of bias related to the studied population and high prevalence of gastric cancer metastases in Asian patients compared to Western population with dominant breast cancer metastases or melanoma. The authors strongly suggest looking for predictive factors and analyzing outcomes of specific types of metastases even for rarely reported cases. It has to be done with multi-institutional studies. Sano et al. did not show specific outcomes of laparoscopic approach in this group of patients.

Some of the above mentioned prognostic factors are related to the technique of liver resection such as major hepatectomy. Our group has worked with the parenchyma-sparing concept for LLR since the late 1990s [13, 33, 34]. It seems that the parenchyma-sparing concept, enabling sparing of functional capacity and facilitating potential re-resections of the liver, could be as effective for NCNNLM as it is for CRLM [13, 34, 35]. Despite the skepticism towards this technique in regard to oncological radicality, it has been shown that the parenchyma-sparing for CRLM does not compromise oncologic outcomes [16, 34]. In current series, major hepatectomy was done in three patients and parenchyma-sparing resections in remaining 48 cases. Fourteen patients (27%) in our series received R1 resection; however, local recurrence in the resection bed was observed in only one (Table 4). This can be explained by the use of energy-based surgical instruments for parenchyma transection that induces thermal damage to the surrounding tissue and thus creates an additional zone of tissue necrosis [13]. It appears that in selected cases parenchyma-sparing liver resection can be combined with ablation to avoid major resections as it is described for CRLM [36]. In the current study, seven patients (14%) underwent liver resection with concomitant radiofrequency ablation (Table 3).

Survival is mainly dependent on the type of primary tumor and on factors related to patient characteristics and aggressiveness of the disease (Table 5). The prognostic model proposed by Adam et al. [7] was validated in previous studies [29, 37, 38]. However, since the introduction of the model, there has been more focus on patient selection which resulted in further variability of populations between studies. Some authors excluded metastases of sarcomatous origin intentionally, considering them as a well-defined indication for liver resection with good outcomes [21, 39, 40]. However, different subtypes of sarcomas should be assessed separately as their behavior is different [41]. Patients with liver metastases from GISTs have better survival associated with the effectiveness of the tyrosine kinase inhibitor sunitinib [1, 42].

In our series, we found 16 patients (31%) with stable or resectable extrahepatic disease (EHD). These patients are frequently not considered for surgery [39]. In Adam’s study, EHD was not a contraindication for surgery but patients with extrahepatic metastases had worse survival compared to patients with metastatic disease limited to the liver. This was confirmed in the studies that accepted patients with EHD for liver resection where EHD was an independent predictor of reduced OS and RFS [43]. However, exclusion of patients with any EHD may result in missing treatment opportunity in some of these patients. Diseases-specific approach and a more restrictive selection of patients based on analysis of response to preoperative chemotherapy may influence both the decision for liver resection and postoperative long-term outcomes. Gandy et al. [44] suggested a better selection could be achieved by extending the preoperative assessment period by combining chemotherapy regimens with local therapy. A better understanding of tumor biology may be a key to successful treatment as was shown in case of RAS mutations in colorectal cancer [45, 46] and estrogen (ER), progesterone (PR), and HER2 receptor status in breast cancer [1, 47].

Selection of patients for NCNNLM group is based on a common site of metastases and exclusion of well described groups of colorectal and neuroendocrine metastases. Thus, the analyzed population is very heterogeneous. The current analysis may aid in selection of patients for surgery but have to be approached with caution because it is a substitute for more accurate analyses of individual types of tumors. These are unlikely to be accomplished for most of the types of NCNNLM due to low numbers of patients being referred for surgical treatment. Our institution is the only performing liver surgery in our region, and this minimizes any bias based on referral patterns. In this study, a control group is missing and we tried to compensate this by comparing our data with available literature on open liver surgery for NCNNLM. The retrospective design is another limitation of our study.

## Conclusions

Laparoscopic liver resection for NCNNLM results in good short-term outcomes comparable to other indications for laparoscopic hepatectomy. In specific groups, long-term outcomes may be comparable to those reported for colorectal metastases. Laparoscopic approach should be considered in patients selected for surgical treatment. Patient selection for surgery is of utmost importance in this population based on patient and primary tumor characteristics.

## Data Availability

The data used for the current study is available from the corresponding author on reasonable request.
